# Cardiovascular Risk in Children: Focus on Pathophysiological Aspects

**DOI:** 10.3390/ijms21186612

**Published:** 2020-09-10

**Authors:** Simonetta Genovesi, Gianfranco Parati

**Affiliations:** 1 School of Medicine and Surgery, University of Milano - Bicocca, 20100 Milan, Italy; gianfranco.parati@unimib.it; 2 Istituto Auxologico Italiano, IRCCS, Cardiologic Unit, 20100 Milan, Italy

**Keywords:** cardiovascular risk, children, cytokines, dyslipidemia, obesity, insulin resistance, nitric oxide, uric acid, visceral adiposity, vitamin D

## Abstract

Cardiovascular diseases are the leading cause of death, disability, and health care costs in industrialized countries. In general, cardiovascular diseases occur in adulthood, but cardiovascular damage, including stiffening of the arteries, begins very early. Already in the first decade of life, alterations that will favor the formation of atherosclerotic plaques may be present. Cardiovascular risk factors, associated with genetic predisposition, may trigger a sequence of pathophysiological changes which are associated with the progression of the atherosclerosis process. In this frame, the role of obesity has been increasingly emphasized. Different mechanisms linking obesity to cardiovascular disease have been postulated. Endothelial dysfunction and subclinical inflammation seem to be related to the worsening of cardiovascular risk factors in obese subjects and might have an essential role in the development of insulin resistance and the initiation and progression of atherosclerotic lesions. Excess weight, and in particular visceral adiposity, are associated with hypertrophy and hyperplasia of the adipocytes, increased secretion of adipokines and inflammatory cytokines and increase in serum uric acid levels. The list of obesity-related biomarkers associated with cardiovascular damage is rapidly expanding and their importance has already been described in children as well. Pathophysiological changes involved in determining early cardiovascular damage starting from childhood are discussed in this Special Issue.

Cardiovascular diseases are the leading cause of death, disability and health care costs in industrialized countries. While cardiovascular diseases generally become clinically manifest in adulthood, damage to the cardiovascular system, including stiffening of large arteries, may start very early in life. Already in the first decade of life, alterations that will lead to formation of atherosclerotic plaques may be present. A number of cardiovascular risk factors are associated with the progression of the atherosclerotic process starting early in life. Figure 1 shows the main mechanisms that can induce early cardiovascular damage in children.

The presence of early atherosclerosis is an alarming phenomenon that was demonstrated several years ago by the findings of the Bogalusa Heart Study [1]. Observations from post mortem biopsy studies in children aged between 2 and 15 years showed that nearly all of the children presented fatty streaks in the aorta and that the prevalence of fatty streaks in the coronary arteries was approximately 50%. In addition, the prevalence of fibrous-plaques was around 20% at the aortic level and 8% at the coronary level. The extent of the lesions in the aorta and coronary arteries was strongly associated with higher body mass index (BMI) and blood pressure values, and with alterations in the lipid profile. These data are particularly worrying, especially in consideration of the important increase in the prevalence of obesity in children that has occurred in the last decades. Undoubtedly, some of the causes of this phenomenon are incorrect lifestyle and wrong eating habits that begin as early as in childhood. In the United States, between 2011 and 2014, the prevalence of hypercholesterolemia in the 6–19 age group was estimated to be 7.4%, with even higher percentages in obese subjects [2]. It was demonstrated that obesity and the metabolic syndrome in children can lead to dyslipidemia and early vascular disease [3]. The vascular lesions that begin at a young age and that will lead to atherosclerosis are mainly located in those segments of the arterial tree where the blood flow is not lamellar. Hemodynamic forces act on the endothelial cells and alter their permeability, modifying the expression of endothelial cell genes such as that for nitric oxide synthase (NOS) and, consequently, the production of nitric oxide (NO). This situation favors the infiltration of the arterial wall by low-density apolipoproteins which undergo oxidation phenomena, causing an inflammatory state that attracts monocytes which, in turn, trigger other inflammation factors, thereby causing a vicious circle. The monocytes subsequently turn into macrophages which, by accumulating lipid materials, turn into foamy cells, thus starting the actual lipid accumulation characteristic/process of the atherosclerotic plaque [4]. Both the initiation and the maintenance of these processes are favored by some associated predisposing factors: arterial hypertension, quantitative and qualitative alterations of low-density apolipoproteins (in particular an increase in low-density lipoproteins (LDL) cholesterol and lipoprotein a), hyperglycemia, hyperuricemia and environmental factors such as exposure to smoke and pollution. High-density lipoproteins (HDL) may have a protective function not only through the removal of cholesterol from the vessels, but also by their well-known anti-inflammatory function that counteracts the processes described above. Therefore, low HDL values should be considered a risk factor for atherosclerosis [5]. Increased plasma levels of fatty acids (FA) can also constitute a cardiovascular risk factor, starting as early as in pediatric age. Indeed, an association was observed between increased levels of saturated FA and metabolic syndrome in obese children [6]. The data of Bonafini et al. [7] supported the role of FA metabolism alterations in this setting. Their study confirmed that, on average, Italian children display low levels, about 4%, of omega-3 FA (an unsaturated FA), reflecting modest dietary intake of unsaturated FA, and overweight children show an even lower level. No clear conclusion about the effects of another unsaturated FA, omega-6 FA, can be drawn from the study, but the results confirm that delta-6 desaturase activity is associated with adiposity/metabolic indices, in particular waist/height ratio, glucose and cholesterol, especially in Caucasian children. An unexpected observation regards palmitoleic acid, an omega-7 monounsaturated FA, which is not substantially influenced by dietary intake, but that derives from the metabolism of palmitic acid by stearoyl-CoA desaturase-16 (SCD-16). In the Bonafini study, this FA and the estimated SCD-16 activity itself were associated with markers of adiposity (BMI, waist/height and fat mass) and blood pressure. Again, the effect was more evident in the excess-weight group and in Caucasian children. This association could reflect a genetically driven increased activity of the SCD-16 rather than a deleterious effect of palmitoleic acid per se, but the fact that the association was stronger in overweight/obese could also suggest that other environmental (obesity) or dietary (excess intake of the precursor, palmitic acid) factors can sustain the association, leading to a metabolic reaction.

Endothelial hyperpermeability, a major factor favoring intimal LDL uptake, is an early step of atherogenesis which is accelerated in the presence of disorders of the glucose metabolism. Little is known about the causal links between hyperglycemia and endothelial leakage. With the aim to provide new information on this point, Cazzaniga et al. [8] loaded sera from pediatric type 1 diabetes mellitus patients on primary human endothelial cells. They showed that permeability was enhanced only by hyperglycemic sera and that this hyperpermeability was prevented by genetic or pharmacological inhibition of inducible NOS. Because these results were phenocopied by culturing endothelial cells in high glucose, it is feasible to conclude that high extracellular glucose directly impairs the endothelial barrier through an increased synthesis of nitric oxide. The authors concluded that to translate these results into a clinical setting the importance of tightly controlling glycemia and preventing also sporadic transitory peaks should be underlined. In addition, countermeasures that decrease inducible NOS should be considered, like aerobic exercise which significantly decreases inducible NOS in pre-diabetic animal models. However, the results of the study by Cazzaniga et al. [8] are not in agreement with the findings by Hsu et al. [9] in pediatric patients with chronic kidney disease but without alterations of glycidic metabolism, that show an association between altered blood pressure parameters, lower arginine levels (the substrate for NOS for the generation of NO) and higher values of the citrulline-to-arginine ratio. These discordant findings demonstrate that the interactions between NO and cardiovascular risk factors in children are complex and different in various clinical situations and that they deserve further investigation. The presence of both type 1 and type 2 diabetes mellitus, which is becoming more frequent even in children (especially type 2), is another important cardiovascular risk factor. In their review, Pastore et al. [10] described how hyperglycemia in diabetic children is not the only factor favoring the onset of cardiovascular diseases, but that other risk factors, only partially modifiable, contribute to this process as well. Among these, a central role, in particular with regard to type 2 diabetes mellitus, is played by the presence of obesity which, even in childhood, is frequently associated with insulin resistance and hypertension [11,12]. It should be noted that this cluster of risk factors (excess weight, hyperinsulinism and hypertension) is often present even in severely overweight children who do not show overt diabetic disease [13]. In addition to the presence of obesity, it is important to consider the distribution of fat mass. In children, as in adults, a higher waist circumference with the same BMI z-score represents an additional cardiovascular risk factor [14,15]. A negative correlation between adiponectin (a cytokine that has anti-inflammatory, anti-atherogenic and insulin-sensitizing properties) and waist circumference has been described in children [16,17,18]. Furthermore, high levels of leptin, a cytokine largely secreted by adipose tissue, are associated with biomarkers of cardiovascular risk already at a pediatric age [19]. The measurement of body fatness and fat distribution for the estimation of cardiovascular risk in pediatric age is therefore of critical importance and widespread agreement has been achieved to use it in daily clinical routine. However, its feasibility in practice is still difficult. New imaging techniques for body composition assessment have been proposed in the last few decades (i.e., magnetic resonance imaging, computed tomography, and dual-energy X-ray absorptiometry). These techniques are more precise but too expensive and time-consuming, and thus not suitable to be applied (at bedside) in the pediatricians’ office. This is the reason for a renewed interest for anthropometry as a simple, portable and costless technique. Leone et al. [20] compared the relationship between parameters of the metabolic syndrome and some anthropometric adiposity indices in obese children and adolescents, including BMI, waist circumference and the recently proposed body shape index (ABSI) [21], obtained by a mathematical combination of waist circumference, BMI and height. They found that the inclusion of ABSI in prediction models for metabolic syndrome improved the prediction compared to BMI alone in children >10 years of age and concluded that the combined ABSI-BMI can be a useful index for evaluating the relative contribution of central obesity to cardio-metabolic risk in the clinical management of obese children and adolescents. Anthropometry has a long history. BMI was suggested by Quetelet in 1832 [22], while waist circumference percentiles were first proposed in 2001 by McCarthy [23]. Anthropometry thus seems to take its revenge on modern body composition techniques thanks to its simplicity. The overall estimation of the risk for cardio-metabolic and vascular diseases, however, requires a complex evaluation of several parameters at a pediatric age, visceral obesity being only one piece of the puzzle.

In recent years, some non-traditional cardiovascular risk factors associated with obesity have become increasingly evident. In childhood, excess weight is often associated with an increase in plasma uric acid values and hyperuricemia must be considered a cardiovascular risk factor even in pediatric age, mainly due to its association with the development of insulin resistance and hypertension [24]. In their article, Russo et al. [25] reviewed how fructose and uric acid begin to mediate the risk of cardiovascular disease from the first decades of life. In particular, their combined effects on insulin resistance and its correlates (such as obesity, diabetes, hypertension and non-alcoholic fatty liver disease) contribute to the worsening of cardiovascular risk in a decisive way. According to Russo et al. [25], a role for fructose and uric acid as mediators of kidney injury is also supported by experimental and clinical studies. Discussion of the biological mechanisms underlying these associations highlight how the interplay between fructose, uric acid, activation of the renin angiotensin aldosterone system and pathways of inflammation is a strong inductor for the development of clinical conditions at increased cardiovascular risk. Children can be considered extraordinary models for studying the mechanisms of disease development, as potential confounding factors linked to co-morbidities or to the physiological aging are usually absent in the very early years of life. It has been proposed that the pathogenetic mechanisms linking fructose and serum uric acid to the development of these high-risk conditions may follow a biphasic pattern: an initial functional phase followed by an irreversible phase characterized by the development of structural vascular and organ damage [26]. This hypothesis might explain the negative results reported in studies investigating the use of urate-lowering treatments in adults with established hypertension or chronic renal disease.

Another review, perhaps less closely related to the other articles, but not less important, completes this Special Issue on the pathophysiological aspects of cardiovascular risk factors in pediatric age by focusing on the role of Vitamin D. In recent years, cardiovascular effects of vitamin D have been gaining interest. The vitamin D pleiotropic effects are widely recognized and studied; however, the mechanisms by which vitamin D exerts its cardio- and vasculoprotective effects are not fully understood yet. Certainly, it is a powerful modulator of inflammation through different pathways. In vitro, vitamin D appears to suppress the intracellular nuclear factor κB (NF-κB) pathway and renin synthesis, thereby attenuating the progression of coronary artery disease. In recent years, new studies have been performed also in pediatric populations. The studies, however, have yielded conflicting data. Savastio at al. [27] addressed the topic of the relationship between vitamin D and cardiovascular risk in children. An update on these issues in pediatric patients is certainly of interest to focus on the possible benefits of vitamin D supplementation. The review by Savastio et al. [27] provided a summary of the actual knowledge of the pathophysiologic role of vitamin D in cardiovascular disease. Moreover, the authors discussed whether vitamin D supplementation may influence cardiovascular risk markers by its anti-inflammatory properties. Several observational studies evidenced an inverse correlation between low levels of vitamin D and cardiovascular risk factors in children, in particular increased blood pressure and worse lipid profile. These studies support the thesis that increased risk of cardiovascular diseases occurs primarily in people with vitamin D deficiency. Therefore, in recent years, new randomized controlled trials examining the effects of vitamin D supplementation on cardiovascular outcomes in children and adolescents have been performed. Most randomized controlled trials (RCTs) have demonstrated that cardiovascular risk markers, such as lipid parameters, inflammation markers, blood pressure and arterial stiffness are unaffected by vitamin D supplementation. Overall, the results of RCTs did not support vitamin D supplementation for reducing cardiovascular risk in children and adolescents, in accordance with studies conducted in adults. By contrast, a slight improvement of cardiovascular markers was found in two RCTs in obese adolescents with baseline mean vitamin D deficiency. Moreover, a recent meta-analysis showed how insulin resistance decreased in overweight/obese children and adolescents after vitamin D supplementation. Further standardized supplementation studies are needed to assess the benefit of vitamin D supplementation in children and adolescents at increased risk of cardiovascular diseases, such as obese patients.

In conclusion, several factors contribute to increasing the cardiovascular risk in children and there are numerous and complex interactions between them. Many aspects are not yet fully elucidated, but it is certainly clear that identifying children with cardiovascular risk factors in order to start prevention as early as possible is fundamental for their health and for preventing them from developing cardiovascular diseases in their adult lives.

## Figures and Tables

**Figure 1:**
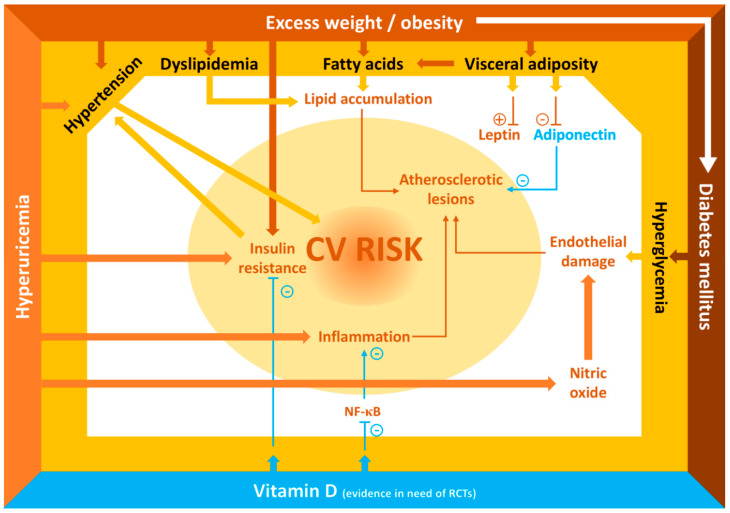
Main cardiovascular risk factors in childhood. Abbreviations: CV, cardiovascular; NF-κB, nuclear factor κB, RCTs, randomized controlled trials.

## Abbreviations

ABSI	Body shape index
BMI	Body mass index
FA	Fatty acids
HDL	High density lipoproteins
LDL	Low density lipoproteins
NF-κB	Nuclear factor κB
NO	Nitric oxide
NOS	Nitric oxide synthase
RCT	Randomized Controlled Trial
SCD-16	Stearoyl-CoA desaturase-16
